# An Infectious Silver Lining: Is There a Positive Relationship Between Recovering From a COVID Infection and Psychological Richness of Life?

**DOI:** 10.3389/fpsyg.2022.785224

**Published:** 2022-04-25

**Authors:** Micael Dahlen, Helge Thorbjørnsen

**Affiliations:** ^1^Stockholm School of Economics, Stockholm, Sweden; ^2^Norwegian School of Economics, Bergen, Norway

**Keywords:** psychologically rich life, COVID, surveys, wellbeing, exploratory, good life

## Abstract

This paper draws from the recent literature on psychological richness of life (PRL), conceptualized as a third dimension of a good life which would be particularly desirable when happiness or meaning in life cannot be satisfactory attained, to investigate whether recovering from a COVID infection could be associated with PRL. We hypothesize that people who have recovered from being infected by the virus rate their PRL higher than those who have not been infected. Two cross-sectional studies (*n* = 937, and *n* = 1,012) support the hypothesis, and also found that people who recovered from a COVID infection were less prone to want to delete the pandemic time period from their life line and reported lower levels of death anxiety. The findings have implications for coping both on a societal and individual level, by changing perspectives and valuing the richness of positive as well as negative experiences, as well as counteracting repetitiveness and tedium and stimulating new experiences and reflection. The findings also have implications for future research on well-being, which could be informed by expanding the perspective from living well to a life well-lived, and future research on PRL and coping in terms of investigating causalities and interaction effects.

## Introduction

Most people want life to be happy or meaningful (Oishi et al., 2020). However, research suggests that these dimensions of life have been particularly hard to achieve during the COVID pandemic. Recent studies have found that happiness has decreased on average compared to before the pandemic (e.g., Greyling et al., 2021; Lin et al., 2021; Rossouw et al., 2021; VanderWeele et al., 2021). There is a growing body of research on how quarantine-related factors, such as boredom (Brooks et al., 2020), decreased physical activity (e.g., Dahlen et al., 2021), work-life boundary-blurring (Pluut and Wonders, 2020), loneliness (e.g., Stieger et al., 2021), and financial worry (e.g., Bono et al., 2020), have all been found to impact negatively on people’s happiness, as have, of course, fear of and worry about being infected by the virus (e.g., Lee et al., 2020). Recent studies have also found average perceptions of meaning in life to be lower during the pandemic compared to before (e.g., Arslan and Yıldırım, 2021; Chasson et al., 2021; Yıldırım and Güler, 2021). A growing body of research details how pandemic-related factors, such as shattered goals (e.g., de Jong et al., 2020), inability to make best use of one’s time (Martinelli et al., 2020), and stress (e.g., Schnell and Krampe, 2020), impact negatively on people’s perceived meaning in life.

However, the recent literature suggests that there is a third dimension of a good life, alongside happiness and meaning, conceptualized as a psychologically rich life (e.g., Besser and Oishi, 2020; Oishi and Westgate, 2021). This dimension is defined as a life characterized by a variety of interesting and perspective-changing experiences (Oishi and Westgate, 2021), which need not be positively valenced and can even be unpleasant, to be contrasted with a boring and monotonous life defined by routines that just are not that interesting (Besser and Oishi, 2020). While less desirable than a happy or meaningful life, recent research has found that psychological richness does indeed contribute to people’s perceptions of a good life (Oishi et al., 2020) and would be particularly valuable when happiness and meaning cannot be achieved (Oishi et al., 2019).

Contrary to the other two dimensions, the psychological richness of life (PRL) has not yet been subject to study during the recent pandemic. This is the aim of the present paper. More specifically, it focuses on the relationship between recovering from a COVID infection and PRL. We believe that a COVID infection would fit the definition of a psychologically rich experience. Not previously experienced in our lifetime, unpredictable, and with a multitude of potential symptoms (e.g., Norton et al., 2021; Tsang et al. 2021), it would likely be a dramatic and perspective-changing experience and upset any boredom and monotony imposed by the pandemic. The question seems highly relevant, considering the fact that this experience would be potentially shared by more than 260 million people worldwide who have recovered from a COVID infection to date (WHO, 2022, Worldometers 2022).

We test our main hypothesis that there is a positive relationship between recovering from a COVID infection and psychological richness in life, in two cross-sectional online studies. In study 1, a convenience sample of 937 Swedes, we also investigated perceived PRL change and whether people who recovered from a COVID infection would be less prone to erase the pandemic time-period from their lives (and explored their underlying motivations for this decision). In study 2, a survey of a nation-representative sample of 1,012 Swedes, we replicate the findings from study 1 and also test the hypothesis that people who recovered from a COVID infection experience less death anxiety.

## Theoretical Framework: A Good Life and Psychological Richness

A survey of almost 4,000 people in nine different countries in North America, Asia, Africa, and Europe, found that between 50 and 70% percent would describe their ideal life as happy, while between 25 and 38% would describe it as meaningful (Oishi et al., 2020). Happiness and meaning have been subject to study as two dimensions of well-being for decades (for a review, see Diener et al., 2018). Happiness represents a dimension of hedonic or subjective well-being (e.g., Diener et al., 1999), and meaning in life represents a dimension of eudaimonic or psychological well-being (e.g., Ryan and Deci, 2001), whereas well-being is conceptualized as optimal psychological experience and functioning (Deci and Ryan, 2008), or the feeling or belief that life is going well (Diener et al., 2018).

However, optimal psychological experience and functioning might not be possible for everyone to attain (e.g., Lachman and Weaver, 1998; Wrosch et al., 2003). Circumstances might not allow for a happy life, characterized by stability, safety, comfort, and pleasantness (e.g., Oishi et al., 2019; Besser and Oishi, 2020). Similarly, a meaningful life, characterized by purpose, structure, and the exercise of one’s capacities (e.g., Oishi et al., 2019; Besser and Oishi, 2020), might also not be allowed by circumstances. In lack of happiness and meaning, can people still perceive that they live a good life?

Conceived of as a life well lived (Oishi and Westgate, 2021), a good life expands on the definition of well-being to allow for the possibility of living a desirable life even when life is not going well. A good life would be possible to attain not only by way of happiness or meaning, but also when life is rich with experiences that are not necessarily pleasant or purposeful. Indeed, the survey by Oishi et al. (2020) found that between 9 and 17% percent of people would describe their ideal life in terms of what the authors conceptualize as a psychologically rich life, characterized by a variety of challenging, surprising, dramatic, and perspective-shifting events (e.g., Oishi et al., 2019; Besser and Oishi, 2020).

As argued by Besser and Oishi (2020), the nice thing about a psychologically rich life (PRL) is that, conversely to a happy life or a meaningful life, it is an accessible one which can be attained in a number of different ways. Willingly and deliberately, but also unwillingly and not deliberately (Oishi and Westgate, 2021). For example, a 14-day diary study found that people who had made some kind of excursion or spontaneous trip during the time period rated higher on PRL, and a study comparing students taking a semester abroad vs. staying on campus found a positive correlation between the former groups higher frequency of novel unusual experiences and their higher PRL ratings (Oishi et al., 2020b). In a survey of people who had just exited an escape room, those who managed to “escape” rated higher on happiness and meaning than those who failed, but their PRL ratings were unrelated to the outcome (Oishi and Westgate, 2021). Instead, PRL ratings increased with the perceived difficulty (and likelihood of *not* escaping). Oishi and Westgate (2021) also report on a series of studies where coders who were blind to the hypotheses rated obituaries higher on PRL (while simultaneously lower on happiness) when they contained drama and misfortunes.

Indeed, a psychologically rich life could be construed as a good life, even lacking happiness and meaning, when a person would be able to say on their deathbed, “I had an interesting life,” or that their life would make a good novel or movie (Oishi et al., 2019).

It is well established that the two dimensions of well-being, happiness, and meaning, overlap to some extent, but are still unique and discriminantly valid constructs (e.g., Diener et al., 2018). Similarly, the three dimensions of a good life overlap to some extent but are still unique and discriminantly valid concepts. In the previously mentioned global survey of people’s ideal lives, happiness and meaning correlated in the range of 0.23–0.68 across countries, whereas happiness and PRL correlations ranged between −0.11 and 0.51 and meaning and PRL correlations ranged between 0.34 and 0.59. In a test of their psychometric properties, Oishi and Westgate (2021) found that two-factor models where happiness, meaning, and PRL were combined in different ways produced a goodness of fit in the range of 0.7 to 0.9 across studies, but only the three-factor model (where happiness, meaning, and PRL were separated) produced a goodness of fit in the higher 0.9 s and did so consistently across studies. In a repeated measures study, Oishi et al. (2019) similarly found that the items used to measure PRL had discriminant validity across time versus the other two dimensions.

Applying the three dimensions of a good life to the COVID pandemic, recent studies have found that lockdowns, quarantines, restrictions, and fear of the virus have had detrimental effects on both happiness and meaningfulness, as briefly reviewed in this paper’s introduction. However, PRL has not yet been studied in light of the pandemic. We hypothesize that there is a positive relationship between PRL and recovering from a COVID infection.

## Hypothesis 1: A Positive Relationship Between Recovering From a COVID Infection and PRL

While previous studies have investigated the indirect relationships between happiness, meaning and the pandemic (through restrictions, living conditions, and reactions such as stress) to the best of our knowledge, only one published study has associated well-being directly with the COVID virus. In that study, Dahlen and Thorbjørnsen (2021) found a positive relationship between recovering from a COVID infection and perceived meaning in life. We expect a similarly positive relationship between recovering from a COVID infection and PRL.

A three-fold argument could be made for this hypothesis. First, a COVID infection, unprecedented with its unpredictability and multitude of symptoms (e.g., Norton et al., 2021; Tsang et al., 2021), would fit well with the construal of a psychologically rich experience as challenging, surprising, and perspective shifting, breaking the tedium and monotony of pandemic life, and potentially adding drama and stories to one’s life narrative (e.g., Oishi and Westgate, 2021). Second, perceiving life as psychologically rich could be a way to cope with the infection. This would resonate with previous studies on happiness-mindfulness (e.g., Carreno et al., 2021), meaning-centered coping (e.g., Polizzi et al., 2020; Lin, 2021; Eisenbeck et al., 2022), and psychological flexibility (e.g., Kashdan and Rottenberg, 2010; Arslan et al., 2020; Arslan and Allen, 2021), which have found that people who deliberately impose meaning into the experience, or redefine it as adding some kind of value to life, are more resilient to the negative effects of the pandemic. By this logic, perceiving the infection as psychologically rich would add to, rather than subtract from, a good life, and be particularly necessary in this directly (vis-á-vis indirectly for those not infected) threatening situation. In other words, the positive association could potentially be with both (absolute level) PRL and perceived change in PRL. Third, as PRL and meaning are overlapping to some extent, and perceived meaning in life has been found to have a positive relationship with recovering from a COVID infection (Dahlen and Thorbjørnsen 2021), we would expect PRL to have a similarly positive relationship with recovering from a COVID infection. Therefore, we hypothesize:

*H1*: People who have recovered from a COVID infection rate their psychological richness of life higher compared to those who have not been infected with the virus.

## Hypothesis 2: A Negative Relationship Between Recovering From a COVID Infection and Proneness to Erase the Pandemic Time-Period

We hypothesize that people who recovered from a COVID infection would be less prone to erase the pandemic time-period from their lifeline, if given the choice, compared to those who have not been infected by the virus. As found in the reviewed studies, the pandemic time period has taken much from people’s lives, for example, in terms of stability, safety, comfort and pleasantness (which would otherwise contribute to a happy life), in terms of purpose, structure and the ability to live up to one’s full capacity (which would otherwise contribute to a meaningful life), and potentially also in terms of experiences and variation due to restrictions (which would otherwise contribute to a psychologically rich life). Consequently, it seems plausible that many would rather erase it from their lifeline. However, compared to those who have not been infected and directly impacted by the virus, those who recovered from a COVID infection would be more likely to perceive that the pandemic time period has enriched their lives, in terms of, for example, challenge, intense experiences, new perspective and a story to their life narrative, and it seems reasonable to expect that they would therefore be less prone to erase it from their lifeline.

In fact, if recovering from a COVID infection has a positive relationship with PRL and PRL is a dimension of good life, which by definition is a life worth living (Oishi and Westgate, 2021), the logical extension would be that the time-period of the COVID infection would be worth keeping on the lifeline. Therefore, we hypothesize:

*H2*: People who have recovered from a COVID infection would be les\s prone to erase the pandemic time-period from their lifeline compared to those who have not been infected with the virus.

## Research Question: Main Motivations for Erasing the Pandemic Time-Period or Not?

While a positive relationship between recovering from a COVID infection and PRL could explain the potential decision to not erase the pandemic time-period from one’s lifeline, it is not conceptually clear why people would rather prefer to erase it. Would it, for example, be because they regret the lower level of PRL, or because other, negative, motivations become more salient in the absence of a higher level of PRL? In other words, are the motivations for erasing the time-period the same as for not erasing it, or do they differ? The answer to this question would inform our understanding both of pandemic coping and of PRL. Therefore, we pose the research question:

RQ1 *What are people’s main motivations for wanting to erase the pandemic time-period or not from their lifeline?*

## Study 1

### Materials and Methods

#### Participants

A survey was distributed on the first author’s Facebook page during the second week of April 2021. A total of 973 Swedes (68.7% females, mean age 55.0 yrs., age span 20–93 yrs.) anonymously filled out the questionnaire. The respondents were informed that they consented to be included in the study by answering the web-based questionnaire. The study was conducted in accordance with the Declaration of Helsinki (World Medical Association, 2013).

#### Measures

People answered “yes” or “no” to the question, *“have you recovered from a diagnosed COVID infection?”* We also included the option, “no, but have started or finished vaccination treatment.”

*PRL,* our main dependent variable (H1), was measured with five items taken from Oishi et al. (2020), “My life, overall, is… eventful/dramatic/interesting/full of surprise/psychologically rich,” on a 10-point scale from 1 (completely disagree) to 10 (completely agree), *Cronbach’s alpha* = 0.83, *McDonald’s Omega* = 0.83.

Per the argument leading up to H2 that the positive association of recovering from a COVID infection could potentially be both with absolute level and with perceived change in PRL, we also gauged people’s *perceived change in PRL* during the pandemic with the question, “During the pandemic, has your life, compared to before, become more or less… eventful/dramatic/interesting/full of surprise/psychologically rich,” on an 11-point scale ranging from −5 (significantly less) to +5 (significantly more) with midpoint 0, *Cronbach’s alpha* = 0.84, *McDonald’s Omega* = 0.85.

In a confirmatory factor analysis, the five *PRL* items loaded on one factor and the five *change in PRL* items loaded on a second factor in a two-factor model, *Chi-square* = 1101.33, *NFI* = 0.86, *RMSR* = 0.069.

We measured whether people were prone to erase the pandemic time-period from their lifeline (H2) with the question, “if you were able to erase the pandemic time-period and everything that happened during this time, would you choose to do so? Yes/no.”

For exploration per RQ1, we also included an open-ended question, which asked participants to motivate their choice whether to erase the pandemic time-period from their lifeline or not. A total of 531 valid responses were analyzed. First, a random subset of 25 (choosing to erase the time-period) plus 25 (choosing not to erase it) responses was reviewed jointly by two research assistants to identify general themes. They agreed on six themes, “negative impact,” “isolation,” “tedium,” “perspective,” “experience,” and “drama.” The first three were associated with the choice to erase the time-period, and the latter three were associated with the choice not to. Based on these categories, the research assistants independently coded all the responses, *Cohen’s weighted Kappa* 0.93, *p* = 0.001. Differences were then resolved through discussion.

### Results

#### Groups and PRL Measures Correlations

Out of the 973 respondents, 146 (18.3%) answered “yes,” and 420 (52.6%) answered “no” to the question whether they had recovered from a diagnosed COVID infection. An additional 226 (28.3%) had started or finished vaccination treatment. We included them as an additional control group. Six respondents (0.4%) were currently infected and were consequently excluded from the analysis. Correlations between the two main PRL variables are reported in Table 1.

**Table 1:** Descriptive statistics and correlation between the two PRL variables, Study 1.

	*n*	*M*	*SD*	PRL	PRL change
PRL	960	5.79	1.67	–	
PRL change	960	−0.85	1.85	0.45^**^	–

** *< 0.001.*

#### Covariate Analysis

Before testing the hypotheses separately, we first ran a MANCOVA with COVID infection (recovered vs. not infected) as a factor, PRL and perceived PRL change as dependent variables, and including age and sex as covariates. The factor produced significant effects on both dependent variables while controlling for age and sex, *F*(2,553) = 8.10, *p* < 0.001. Next, we subjected the hypotheses to t-testing.

#### Hypothesis Tests

Testing our hypotheses, we conducted mean comparison t-tests between the group that had recovered from a COVID infection and the group who had not had an infection (please refer to Table 2). In addition to our hypothesis tests, we also compared with the group that had started or finished vaccination, for illustrative purposes. As the groups are of unequal sizes, we included nonparametric Mann–Whitney U tests for robustness, reported in Table 3.

**Table 2:** Differences in PRL and propensity to erase the pandemic time-period (mean comparison t-tests, Study 1).

	Recovered from COVID infection		Not infected		*t*	*p*	*Cohen’s d*	Started or finished vaccination		*t*	*p*	*Cohen’s d*	*Chi-square*
	*M*	*SD*	*M*	*SD*				*M*	*SD*				
PRL	6.28	1.52	5.66	1.74	4.05	0.001	0.38	5.84	1.63	2.64	0.005	0.28	
PRL change	−0.32	1.99	−0.90	1.83	3.10	0.001	0.30	−0.97	1.77	3.17	0.001	0.35	
Proportion who would erase pandemic time-period from their lives	15.0%		46.9%					37.4%					15.20

**Table 3:** Differences in PRL (Mann–Whitney U tests, Study 1).

	Recovered from COVID infection		Not infected		*U*	*z*	*p*	Started or finished vaccination		*U*	*z*	*p*
	*n*	*Mdn*	*n*	*Mdn*				*n*	*Mdn*			
PRL	146	6.20	420	5.80	24610.50	−3.56	0.001	226	6.00	14034.50	−2.44	0.008
PRL change	146	−0.20	420	−0.80	25060.00	−3.26	0.001	226	−0.80	13213.00	−3.13	0.001

*Mdn = median.*

#### PRL (H1)

In support of our H1, those who had recovered from a COVID infection rated their PRL significantly higher (*M* = 6.28) than those who had not been infected (*M* = 5.66), *t* = 4.05, *p* < 0.01 (Cohen’s *d* = 0.38). The COVID infection group also rated significantly higher on PRL compared to those who had started or finished vaccination (*M* = 5.84), *t* = 2.64, *p* < 0.01 (Cohen’s *d* = 0.28). Nonparametric Mann–Whitney U tests produced similarly significant differences (Table 3).

The same pattern materialized when comparing people’s perceived change in PRL during the pandemic. While overall negative, those who had recovered from a COVID infection rated their perceived change in PRL significantly less negative (*M* = −0.32, not significantly different from 0 at *t* = 1.95, *p* > 0.05) than those who had not been infected (*M* = −0.90, *t* = 3.10, *p* < 0.01, Cohen’s *d* = 0.30). They also rated higher than those who had started or finished vaccination (*M* = −0.97, *t* = 3.17, *p* < 0.01, Cohen’s *d* = 0.35). Again, Mann–Whitney U tests produced similarly significant differences (Table 3).

#### Proneness to Erase the Pandemic Time-Period From the Lifeline (H2)

In support of H2, those who had recovered from a COVID infection were significantly less prone to erase the pandemic time-period from their lifeline (*15.0%*) than those who had not been infected (*46.9%*). This was also the case when comparing with those who had started or finished vaccination (*37.4%*), *Chi-square* (2, 771) = 15.20, *p* = 0.002.

Testing the argument leading up to H2 that a positive relationship between recovering from a COVID infection and PRL would extend to erasing the pandemic time-period or not, we ran an interaction test between this variable and PRL change (above vs. below 0). Indeed, when PRL change was positive versus negative, significantly more people would choose to keep the time-period on their lifeline and *not* erase it (82.7% vs. 53.8%), *Chi-square* (1, 771) = 52.49, *p* < 0.001.

#### Exploratory Analysis of Motivations to Erase the Pandemic Time-Period or Not (RQ1)

Table 4 summarizes the main themes that were found in the open-ended responses related to why people would choose to erase the pandemic time-period from their lifeline or not (RQ1).

**Table 4:** Motivations to erase the pandemic time-period or not from one’s lifeline, Study 1.

Erase pandemic time-period	Yes	No
Themes	Negative impact (44%) *“Lost my job” “So many people have died” “Many bad things have happened because of it”*	Perspective (41%) *“Gave me time and reason to reflect” “I have learned much about myself” “Changed my outlook on many things”*
	Isolation (20%) *“Isolation and loneliness” “Haven’t been able to meet friends and family” “No social life”*	Experience (38%) *“A life experience” “I experienced things I never thought I would” “It has enriched me in some ways”*
	Tedium (16%) *“Life has been put on pause” “Cannot travel or do anything” “Boring!”*	Drama (11%) *“Life is less boring when it does not stick to the same old track” “Scary but also exciting to see what happens” “It shook things up for me”*
	Other (20%) *“Some things were good, but the bad things outweigh” “Other bad things happened in my life” “Sick of reading all the doomsday news”*	Other (10%) *“I know it was bad for others, but made my life seem less gloomy” “I do not believe in regrets” “There’s no changing history”*

The main themes that were recurrent in explaining the choice to erase the pandemic time-period from one’s lifeline were its negative impact (44%, in terms of losses of, for example, one’s job, the ability to do various things, and even lives), isolation (20%, from everyday life, friends, family, and social contexts), and tedium (16%, with nothing to do or repetitiveness). These themes came in multiple variations and overall seem to revolve around what the pandemic has taken from life.

The main themes that were recurrent in explaining the choice to *not* erase the pandemic time-period from one’s lifeline were perspective (41%, in terms of, for example, reflecting, learning, and changing one’s outlook), experience (38%, unexpected, unique, enriching), and drama (11%, for example, taking life off track and shaking things up). These themes also came in several different variations and overall seem to revolve around what the pandemic has added to life.

The coders also noted that the replies related to erasing the pandemic time-period often seemed more general (particularly the negative impact theme, which often included references to society and others), whereas those related to not erasing often seemed more personal (references to oneself in all three themes, but particularly the experience and drama themes). Testing this notion, the replies were coded as general or personal (or non-specified) and subjected to a Chi-square test. General references were more common in the replies related to erasing the pandemic time-period (49%) than in the replies related to not erasing (34%), whereas the opposite pattern was found for personal references (41% vs. 55%), *Chi-square* (1, 531) = 25.35, *p* < 0.001.

### Discussion

In support of our main hypothesis, people who had recovered from a COVID infection rated higher on PRL compared to those who had not been infected by the virus (and also in comparison with those who started or finished vaccination). Adding a measure of perceived PRL change, we found that the latter two groups on average perceived that their PRL had decreased during the pandemic, whereas those who had recovered from a COVID infection on average perceived their PRL to remain the same.

In support of our second hypothesis, those who had recovered from a COVID infection were significantly less prone to delete the pandemic time-period from their lifeline than the group that had not been infected. An additional test showed an interaction effect with PRL change, whereby those who experienced an increase in PRL were more prone to keep the time-period compared to those who experienced a decrease in PRL.

The main themes that emerged in our exploratory analysis of the open-ended answers seemingly resonate with the findings. Those who would rather *not* delete the pandemic time-period from their lifeline, generally associated it with perspective, experience and drama, themes that would presumably increase PRL. Those who, on the other hand, would rather delete the pandemic time-period from their lifeline, generally associated it isolation and tedium, themes that would presumably decrease PRL. But the major theme in this group, negative impact, did not relate to PRL. A potential explanation for this would be that a lower level of PRL made other, negative aspects of the pandemic more salient. Additional coding suggested that the motivations to *not* erase the pandemic time-period seemed to be more related to people’s perceptions of their own lives, whereas the motivations to erase it also related to the general (negative) effects of the pandemic.

While the findings support our hypotheses, the study was limited to a convenience sample recruited *via* social media. We therefore conducted a second study to replicate the test of our main hypothesis on a demographically representative sample that was recruited *via* a national panel, and which enabled us to gauge and include age, sex, level of education and income as covariates. This study also tested the additional hypothesis that those who recovered from a COVID infection would rate lower on death anxiety than those who have not been infected with the virus.

## Study 2

### Hypothesis 3: A Negative Relationship Between Recovering From a COVID Infection and Death Anxiety

Studies across the globe, for example, in the USA (Lee et al., 2020), China (Zhang et al., 2020), Pakistan (Shakil et al., 2022), and Europe (Erquicia et al., 2020), have identified increasing death anxiety as a major issue during the COVID pandemic. An argument could be made that death anxiety is, in fact, the root cause of all other factors related to the pandemic, as restrictions on societal and group levels with all its consequences, and precautions on an individual level with its various effects, fundamentally stem from a fear that people would otherwise die (Pyszczynski et al., 2020). Whether people contract the virus or not, the pandemic comprises a contagion of mortality (Courtney et al., 2020). Research on previous pandemics, such as the H1N1 swine flu (e.g., Prati et al., 2011), SARS (e.g., Fung and Carstensen, 2006), and the Ebola epidemic (e.g., Arrowood et al., 2017), has found similar increases in mortality salience and resulting death anxiety.

We hypothesize that there is a negative relationship between recovering from a COVID infection and death anxiety. In other words, those who recovered from a COVID infection would rate lower on death anxiety than those who have not been infected with the virus. Recovering from being actually infected by the virus would likely be a relief from worry about dying from it. But the construal of PRL as a life well-lived—one that would make for good stories, a good movie, or even a good obituary—also suggests that a higher PRL (which was associated with recovering from an infection in Study 1) would potentially mitigate worry about life ending prematurely, at least to some extent.

This argument would resonate with terror management theory, which postulates that mortality salience is associated with increased needs for self-esteem and literal or symbolic immortality to mitigate death anxiety (e.g., Greenberg et al., 1994; Pyszczynski et al., 1999). The literal immortality coping route would mean simply downplaying or denying the threat (Juhl and Routledge, 2016). The symbolic immortality coping route could manifest in, for example, religious activity (e.g., Vail et al., 2010), curiosity (Fitri et al., 2020), or meaning (e.g., Taubman-Ben-Ari, 2011). An argument could be made that PRL would fit within this route as well, being a life well-lived that would carry on in the form of good stories or even a good obituary. By this token, a positive relationship between recovering from a COVID infection and PRL would also imply a negative relationship between recovering from an infection and death anxiety. Therefore, we hypothesize:

*H3*: People who recovered from a COVID infection rate their death anxiety lower compared to those who have not been infected with the virus.

### Materials and Methods

#### Participants

To replicate our main finding from Study 1, we used a demographically representative sample (48.0% females, mean age 50.8 yrs., age span 18–79 yrs.) that was retrieved from the Novus Sweden panel during the first two weeks of June 2021. Participants (*n* = 1,012) were informed that they consented to be included in the study by answering the web-based questionnaire. The study was conducted in accordance with the Declaration of Helsinki (World Medical Association, 2013). All personal data connections were deleted after the material was collected and were not accessible to the researchers in the present study.

#### Measures

Similar to Study 1, people answered “yes” or “no” to the question, *“have you recovered from a diagnosed COVID infection?”* We also included the option, “no, but have started or finished vaccination treatment.”

For robustness, we used a different measure of PRL in this study, consisting of six items taken from the 12-item Psychologically Rich Life Questionnaire (Oishi et al., 2019). We were only able to use six items for space constraints in the proprietary panel. The two authors and two research assistants rated the items on face validity and the six items with the highest scores were chosen. These were: “To what extent would you rate your life as… psychologically rich/emotionally rich/experientially rich/I have a lot of personal life stories to tell others/would make a good novel or movie/on my deathbed, I’m likely to say, “I had an interesting life,” on a 10-point scale from 1 (completely disagree) to 10 (completely agree), *Cronbach’s alpha* = 0.88, *McDonald’s Omega = 0*.88.

Death anxiety was measured with three items taken from the Death Anxiety Scale (Templer, 1970), “I am afraid to die,” “the thought about death never bothers me” (reverse-coded), “I often think about how short life really is,” on a 10-point scale from 1 (completely disagree) to 10 (completely agree), *Cronbach’s alpha* = 0.76, *McDonald’s Omega = 0*.81.

In a confirmatory factor analysis, the six *PRL* items loaded on one factor and the three *death anxiety* items loaded on a second factor in a two-factor model, *Chi-square* = 660.73, *NFI* = 0.84, *RMSR* = 0.068.

### Results

#### Groups and Covariate Analysis

Out of the 1,012 respondents, 144 (14.2%) answered “yes,” and 606 (59.9%) answered “no” to the question whether they had recovered from a diagnosed COVID infection. An additional 260 (25.7%) had started or finished vaccination treatment. Two respondents (0.2%) were currently infected and were thus excluded from the analysis.

Before testing the hypotheses separately, we first ran a MANCOVA with COVID infection (recovered vs. not infected) as a factor, PRL and death anxiety as dependent variables, and including age, sex, level of education, and income as covariates. The factor produced significant effects on both dependent variables while controlling for age, sex, level of education, and income, *F*(2,735) = 5.07, *p* = 0.007. Next, we subjected the hypotheses to t-testing.

#### Hypothesis Tests

Testing our hypotheses, we conducted mean comparison t-tests between the group that had recovered from a COVID infection and the group that had not had an infection (please refer to Table 5). For illustrative purposes again, we also compared with the group that had started or finished vaccination treatment. As the groups are of unequal sizes, we also conducted nonparametric Mann–Whitney U tests for robustness, reported in Table 6.

**Table 5:** Differences in PRL and death anxiety (mean comparison t-tests, Study 2).

	Recovered from COVID infection		Not infected		*t*	*p*	*Cohen’s d*	Started or finished vaccination		*t*	*p*	*Cohen’s d*
	*M*	*SD*	*M*	*SD*				*M*	*SD*			
PRL	6.77	1.59	6.40	1.87	2.42	0.008	0.21	6.51	1.74	1.53	0.06	0.16
Death anxiety	4.89	2.31	5.43	2.38	2.51	0.007	0.23	5.39	2.37	2.08	0.01	0.21

**Table 6:** Differences in PRL and death anxiety (Mann–Whitney U tests, Study 2).

	Recovered from COVID infection		Not infected		*U*	*z*	*p*	Started or finished vaccination		*U*	*z*	*p*
	*n*	*Mdn*	*n*	*Mdn*				*n*	*Mdn*			
PRL	143	6.83	601	6.40	37508.00	−2.37	0.009	258	6.67	16715.50	−1.56	0.06
Death anxiety	143	4.67	603	5.00	48732.50	2.43	0.008	258	5.00	20986.50	2.15	0.02

*Mdn = median.*

#### PRL (H1)

In support of our main hypothesis, those who had recovered from a COVID infection rated their PRL significantly higher (*M* = 6.77) than those who had not been infected (*M* = 6.40), *t* = 2.42, *p* = 0.008 (Cohen’s *d* = 0.21). The COVID infection group also rated higher on PRL compared to those who had started or finished vaccination (*M* = 6.51), though only marginally significant at *t* = 1.53, *p* = 0.06 (Cohen’s *d* = 0.16). Nonparametric Mann–Whitney U tests produced similar differences (Table 6).

#### Death Anxiety (H2)

Also as hypothesized (H3), those who had recovered from a COVID infection rated significantly lower on death anxiety (*M* = 4.89) than those who had not been infected (*M* = 5.43, *t* = 2.51, *p* = 0.007, Cohen’s *d* = 0.23). The COVID infection group also rated lower than those who had started or finished vaccination (*M* = 5.39, *t* = 2.08, *p* = 0.01, Cohen’s *d* = 0.21). Again, Mann–Whitney U tests produced similarly significant differences (Table 6).

#### Test of Mediation, PRL on Death Anxiety

Testing the notion that a COVID infection would impact death anxiety by way of PRL, we ran the PROCESS mediation test recommended by Hayes et al. (2011) (Model 4, 5,000 bootstrapping samples, 95% confidence interval). Recovering from a COVID infection had a significant direct effect on PRL (0.13, 95% CI: 0.03–0.23) and on death anxiety (0.56, 95% CI: 0.02–1.10), but there was also an indirect effect of recovering from a COVID infection (X) on death anxiety (Y) through PRL (0.04, 95% CI: 0.001–0.10).

## Discussion

Our two studies find a positive relationship between recovering from a COVID infection and PRL, in support of the main hypothesis. People who had recovered from a COVID infection rated both their perceived change in PRL and their absolute level of PRL higher, compared to those who had not been infected by the virus. The finding that the average perceived change in PRL was negative in the latter group, but not in the former, suggests that perceiving life as psychologically rich could be a way of coping with the infection, similar to meaning-centered coping (e.g., Polizzi et al., 2020; Karataş et al. 2021; Lin, 2021; Eisenbeck et al., 2022) and psychological flexibility (e.g., Arslan et al., 2020; Arslan and Allen, 2021). The finding that recovered people also rated their absolute level PRL higher on average suggests that a COVID infection could potentially be construed as a psychologically rich experience (*cf.* Oishi and Westgate, 2021).

In support of the second hypothesis, those who had recovered from a COVID infection were also less prone to erase the pandemic time-period from their lifeline. This finding fits the conceptualization of PRL as a dimension of good life, which, by definition, is a life worth living (Oishi and Westgate, 2021). Our exploratory analysis of the participants’ open-ended replies found that the main motivations for keeping the pandemic time-period on the lifeline centered on ways in which it seemingly added to and enriched life.

In support of the third hypothesis, those who had recovered from a COVID infection reported lower death anxiety. PRL was found to mediate the relationship, suggesting that it could function as a symbolic immortality coping route (e.g., Greenberg et al., 1994; Pyszczynski et al., 1999), similar to how people have been found to use, for example, religious activity (e.g., or search for meaning (e.g., Taubman-Ben-Ari, 2011) to cope with increased mortality salience.

### Implications

Our findings contribute to the growing body of research on well-being during the COVID pandemic. While happiness and meaning during the pandemic have been subject to growing bodies of the literature, PRL has not previously received research attention. While the pandemic, with its many restrictions and indirect effects on people’s lives, may overall decrease the richness of life, similar to how happiness and meaning decrease, the PRL perspective gives reason to study the direct effect of being infected by the virus as an undesired and negative experience that could nevertheless enrich life. Our findings also contribute to the nascent literature on PRL by putting it in the pandemic context, a current and global phenomenon, to which everyone can relate, and which can have both positive and associations with people’s psychological richness of life.

Turning to implications, the notion that a good life can manifest even without happiness and meaning, suggests that there are other aspects of wellbeing to monitor and stimulate, both on societal and individual levels, when stability, comfort, and freedom cannot be promoted or attained. These can be simple things, such as counteracting repetitiveness and tedium and stimulating new experiences and reflection. But it would also include the fundamental realization that undesired and unpleasant events and experiences can be construed as valuable, enriching, parts of life—in hindsight, or even as they unfold.

One important function of a psychologically rich life is to cope with tragedy and negative experiences. By valuing *both* positive and negative life events, obtaining new perspectives on life, people may find value in experiences that are not necessarily positive or meaningful. In the same manner as people value sad or scary movies, negative and stressful life-events such as a COVID infection may foster discovery and learning, combat boredom and provide people with vivid memories and more varied lives.

### Summary and Limitations

In summary, our two cross-sectional online studies find a positive relationship between recovering from a COVID infection and PRL. In the first study, a convenience sample of Swedes recruited *via* Facebook, those who reported having recovered from a COVID infection rated their PRL and perceived change in PRL higher than those who reported that they had not been infected, controlling for age and sex. Moreover, a lower percentage in the former versus latter group reported that they would erase the pandemic time-period from their lifeline if they could. In the second study, a nation-representative panel sample of Swedes, those who reported having recovered from a COVID infection rated their PRL higher and their death anxiety lower than those who reported that they had not been infected, controlling for age, sex, level of education, and income. It is important to note that the cross-sectional and correlational designs of study 1 and 2 do not allow for determining causal effects. The group division was based on participants’ self-reports of having recovered from a COVID infection or of never being infected. PRL and death anxiety were measured at the same (single) time, allowing only a “snapshot view” of the relationship. As PRL is not yet a well-established dimension of a good life, it has not been subject to tracking over time (similar to happiness and meaning), and therefore, it was not possible to obtain pre-pandemic measures for comparison and analysis of dynamic effects. To this end, future studies are needed, using additional methodological approaches such as longitudinal designs or experiments, and preferably using clinical test data rather than self-reports of COVID status. While controlling for age, sex, education, and income, the analyses in the present studies were limited to two small samples of Swedes. Future research would be informed by larger samples, which take potential moderating effects of demographic and socioeconomic variables, as well as national differences into account.

## Data Availability Statement

The datasets presented in this study can be found in online repositories. The names of the repository/repositories and accession number(s) can be found at: DOI 10.17605/OSF.IO/CGM3W.

## Ethics Statement

Ethical review and approval was not required for the study on human participants in accordance with the local legislation and institutional requirements. The patients/participants provided their written informed consent to participate in this study.

## Author Contributions

All authors listed have made a substantial, direct, and intellectual contribution to the work and approved it for publication.

## Conflict of Interest

The authors declare that the research was conducted in the absence of any commercial or financial relationships that could be construed as a potential conflict of interest.

## Publisher’s Note

All claims expressed in this article are solely those of the authors and do not necessarily represent those of their affiliated organizations, or those of the publisher, the editors and the reviewers. Any product that may be evaluated in this article, or claim that may be made by its manufacturer, is not guaranteed or endorsed by the publisher.
